# Reducing the burden of maternal and neonatal infections in low income settings

**Published:** 2011-12

**Authors:** Igor Rudan, Evropi Theodoratou, Harish Nair, Ana Marušić, Harry Campbell

## Abstract

Maternal and neonatal infections remain responsible for up to 1 million deaths each year globally. Current approaches to prevention, early diagnosis and appropriate management are limited by difficulties in developing vaccines against the main pathogens, or alternatively diagnosing the infections accurately and managing them appropriately and effectively in low-resource settings. We propose that short-term priorities should focus on promotion of evidence-based, cost-effective home care practices to prevent maternal and newborn infections, with increased coverage and improved quality of maternal and neonatal care interventions. Longer-term strategic priorities will ultimately need to focus on the development of vaccines and point-of-care diagnostic tests. Diagnostic tests should help establish the aetiological diagnosis and inform treatment decisions. They will also need to be deliverable, affordable, sustainable and acceptable in low-resource settings. The cost-effectiveness of maternal immunization in the protection of neonates will also need to be established.

Millennium Development Goals 4 and 5 require a substantial reduction in child and maternal mortality, respectively, between 1990 and 2015 (1). Infectious diseases are still the major cause of mortality in both population groups. Maternal and neonatal infections remain responsible for more than 1 million deaths each year (2-6), ie, 10-15% of all maternal and child deaths globally. The large majority of these deaths occur in low-income settings, among mothers and children that do not have access to the (underdeveloped) health systems of their countries (2-5). Maternal deaths are clustered around labour, delivery and the immediate postpartum period. HIV/AIDS is a leading cause of death where HIV-related mortality rates are high, and a number of other infectious agents also play a significant role (3). Newborn infections can be divided into early (within the first week of life) and late infections (during weeks 2-4). The former are frequently related to labour and childbirth and are caused by an entirely different spectrum of pathogens than the late neonatal infections (7-9).

Current approaches to prevention, early diagnosis and appropriate management of maternal and neonatal infections globally are limited by difficulties in developing vaccines against the leading prevalent pathogens, or alternatively diagnosing them accurately and managing them appropriately in low-resource settings (4-9). Implementation of existing diagnostic tests and treatments from industrialized countries is challenging in low-resource settings due to their high cost, complexity, infrastructure requirements, inadequately trained end users, low acceptability among health personnel, affected mothers and newborns’ parents, risk of obtaining blood samples for diagnostic testing in unhygienic settings and lack of appropriate quality control measures (5-7).

The information on causal infectious agents in low income settings is available mainly from hospital-based studies, which are not always representative of hospital care at a national level, and may also have limited relevance to settings where most children are born at home (6-8). It is likely that the etiological spectrum also varies significantly across geographic regions (7-9). However, the information on geographic differences in low-income countries is at present very limited. Maternal infections can be caused by a number of bacterial, viral and parasitic agents (2). In neonates, the available data indicate that Gram-negative rods are the major cause in early neonates (the first week of life), where they may cause up to three in every four infections (7,8). *Klebsiella spp*., *Staphylococcus aureus*, *Escherichia coli* and group B *Streptococci* are thought to be the leading causes in the early neonatal period, when most of the deaths occur (7,8). Many of those infections may be environmentally acquired because of unhygienic delivery practices in resource-poor settings rather than being passed on by mothers, which may also explain the predominance of Gram-negative infections among home-born infants (7,8). Their importance decreases in the late neonatal and post-neonatal periods when Gram-positive *cocci* (primarily *Streptococcus*) cause about 2 in every 3 infections (6-9).

Several studies in resource-poor settings have investigated the effectiveness of interventions to prevent and treat maternal and neonatal infections at both community and facility level. It has been reported that skin application of sunflower seed oil provides cheap, safe and effective protection against nosocomial infections in hospitalized preterm neonates and infants (10). Once the infection has developed, the standard treatment approach is oral (for mothers) or parenteral (for newborns) antibiotic treatment. However, a number of very complex and context-specific issues must be considered when selecting the appropriate antimicrobial regimens in the resource-poor settings where most deaths occur. The challenge is to choose a regimen that is effective against the causative pathogen yet affordable in that context, safe for the mothers, foetuses and newborns, and feasible to deliver reliably in the hospital or community setting, as appropriate (11).

Parenteral (intramuscular) regimens for newborns that are currently recommended by the World Health Organization and national paediatric associations comprise a combination of procaine penicillin G (or ampicillin) and gentamicin, or third generation cephalosporins given alone, which are safe and retain efficacy when administered at extended intervals (11). Attempts to estimate the effect of antibiotic use on the reduction of maternal and neonatal mortality in community settings in low income countries have encountered large methodological limitations, but have concluded that all available data suggest a substantial benefit associated with these case management approaches (12). However, recent reports based on hospital-based data suggest alarming rates of laboratory antimicrobial resistance to ampicillin and gentamicin, the first-line antimicrobial agents recommended for the treatment of serious infections in young infants. Significant in-vitro resistance to cotrimoxazole among all the major pathogens and to gentamicin and third generation cephalosporins among *Klebsiella spp*. and emerging resistance in *E coli* are a cause for increasing concern (13).

The strategy promoted by the GAVI Alliance is to save children’s lives and protect their health by increasing access to immunisation in the world’s poorest countries, particularly through acceleration of the uptake and use of underused and new vaccines (14). The successful outcome of this approach is less sensitive to obstacles in accessing health care system throughout the childhood than some other proposed approaches, so it is continuously gaining support and improving child health worldwide. Passive transfer of antibodies from the mother coupled with the immature immune system of neonates acts to reduce the effectiveness of a vaccination strategy in this age period, although this is not true in all cases (eg, maternal tetanus and influenza immunization). Prevention of microbial infection is a priority, because globally, a majority of neonates still die at home, and many of the deaths are thought to be due to infection (2). Maternal immunization probably offers the most promising means of achieving this objective in the longer term. However, vaccines against key pathogens involved in neonatal sepsis are still a long way from final phases of product development and licensing (15,16). It is likely that improving the diagnosis and treatment of neonatal infections will be a central approach to reducing deaths from neonatal infections in the medium term. Therefore, the slow and difficult route through improving local health systems and attention to specific contexts will be required to tackle neonatal infections globally (15,16).

Implementation of existing diagnostic tests and treatments from industrialized countries is challenging in low-resource settings due to their high cost, complexity, infrastructure requirements, inadequately trained end users, low acceptability among health personnel, affected mothers and newborns’ parents, risks associated with obtaining blood samples for diagnostic testing in unhygienic settings, and lack of quality control. We propose that short-term priorities should focus on promotion of cost-effective home-based care practices to prevent maternal and newborn infections, with increased coverage and improved quality of maternal and neonatal care. Longer-term strategic priorities will ultimately need to focus on the development of vaccines and point-of-care diagnostic tests for maternal and neonatal infections. Diagnostic tests should help establish the aetiological diagnosis and inform treatment decisions. They will also need to be deliverable, affordable, sustainable and acceptable in low-resource settings. Cost-effectiveness of maternal immunization in protection of neonates will also need to be established (15,16).

The latter strategy may have different stages. In an earlier stage, tests that could separate viral and bacterial infections, and identify children who need treatment, could be considered a priority for development and implementation. In the longer term, tests that could identify a specific causal pathogen and predict antibiotic resistance may become a focus of interest. The development of such tests should maximize the effectiveness of the chosen treatment, whilst minimizing the emerging problem of antibiotic resistance. Biomarkers are very rarely used in low resource settings, because most of the cases and deaths occur at home and medical laboratories do not even have the most basic facility for blood culture. At this point there is no specific guideline for the use of biomarkers for maternal or neonatal infections. Even if they became available in high-income countries, the biomarkers will not be easily transferable to low resource settings due their high cost and complexity. A new generation of diagnostic tests will be needed at the point-of-care in low resource settings to diagnose neonatal infections, identify responsible pathogens, guide the choice of an appropriate treatment regimen, monitor effectiveness of interventions and determine drug resistance. One of the anticipated uses of the test is also for identifying those neonates that are severely ill and need to be immediately referred to the hospital for intensive care treatment. But in spite of the severe shortage of effective new diagnostics suitable for low-resource settings, there are very few research initiatives to address this problem. This may be due in part to a scarcity of information on the potential health impact and performance of essential diagnostics, and to the low return on investment in diagnostics perceived by the industry (15,16).

Considerable uncertainty still surrounds our current understanding of the epidemiology, aetiology, and effectiveness of available interventions, investment priorities, appropriateness of health policies, and true potential of new preventive interventions and diagnostic tools to address the burden of maternal and neonatal infections globally (2-9). The potential health impact of new diagnostic tools for neonatal infections is uncertain and needs to be modelled based on available information.

In this issue, several papers aimed to review the spectrum of pathogens that threaten maternal and newborn lives in low and middle income countries: Palani Velu et al. focused on maternal bacterial and viral infections (17), Roberts et al. on maternal parasitic infections (18) and Waters et al. on neonatal infections (19). In addition, Saha et al. (20) reviewed the existing biomarkers and diagnostic tests that could be used in low resource settings, while Rubens et al. and Wagner et al. reviewed diagnostic markers that are still in the pipeline and that could be valuable in the diagnosis of neonatal infections, alongside their potential for multiplexing and laboratory requirements (21-22). Understanding the potential impact of new diagnostic tools could encourage donors, researchers, technology developers, policy-makers, international organizations and other stakeholders to enhance their collaboration and focus their efforts, which should in turn lead to a much needed reduction of child deaths from neonatal infections.
